# Estimating cognitive load during self-regulation of brain activity and neurofeedback with therapeutic brain-computer interfaces

**DOI:** 10.3389/fnbeh.2015.00021

**Published:** 2015-02-16

**Authors:** Robert Bauer, Alireza Gharabaghi

**Affiliations:** ^1^Division of Functional and Restorative Neurosurgery and Division of Translational Neurosurgery, Department of Neurosurgery, Eberhard Karls University TuebingenTuebingen, Germany; ^2^Neuroprosthetics Research Group, Werner Reichardt Centre for Integrative Neuroscience, Eberhard Karls University TuebingenTuebingen, Germany

**Keywords:** neurofeedback, cognitive load theory, zone of proximal development, workload, instructional design, brain-computer interface

## Abstract

Neurofeedback (NFB) training with brain-computer interfaces (BCIs) is currently being studied in a variety of neurological and neuropsychiatric conditions in an aim to reduce disorder-specific symptoms. For this purpose, a range of classification algorithms has been explored to identify different brain states. These neural states, e.g., self-regulated brain activity vs. rest, are separated by setting a threshold parameter. Measures such as the maximum classification accuracy (CA) have been introduced to evaluate the performance of these algorithms. Interestingly enough, precisely these measures are often used to estimate the subject’s ability to perform brain self-regulation. This is surprising, given that the goal of improving the tool that differentiates between brain states is different from the aim of optimizing NFB for the subject performing brain self-regulation. For the latter, knowledge about mental resources and work load is essential in order to adapt the difficulty of the intervention accordingly. In this context, we apply an analytical method and provide empirical data to determine the zone of proximal development (ZPD) as a measure of a subject’s cognitive resources and the instructional efficacy of NFB. This approach is based on a reconsideration of item-response theory (IRT) and cognitive load theory for instructional design, and combines them with the CA curve to provide a measure of BCI performance.

## Introduction

Brain-computer interfaces (BCIs) support reinforcement learning of brain self-regulation by feedback and reward. While assistive BCIs aim to replace lost functions by controlling external devices (Yanagisawa et al., 2011; Hochberg et al., 2012; Collinger et al., 2013; Wang et al., 2013), the ultimate goal of restorative or therapeutic approaches is to improve specific functions by neurofeedback (NFB) training, e.g., hand and arm control following a stroke (Ang et al., 2010; Shindo et al., 2011; Buch et al., 2012; Ramos-Murguialday et al., 2013; Gharabaghi et al., 2014a,b). The fundamental approach of NFB is based on the idea that physiological signals during restful waking (Mantini et al., 2007; Albert et al., 2009; De Vico Fallani et al., 2011) are contrasted to the signals during the task condition, using classification algorithms to weight the respective features (Theodoridis and Koutroumbas, 2009). In this regard, restorative BCI is similar to assistive BCI. However, unlike assistive BCI approaches, which select features on the basis of their ability to maximally contrast the two states, the feature space for restorative BCI and NFB training is constrained in accordance with the specific treatment rationale. In stroke rehabilitation, for example, the feature space might be restricted to power in the β-range (15–30 Hz), since decreased movement-related desynchronization in this frequency range is related to the amount of motor impairment after the insult (Rossiter et al., 2014). During restorative BCI training, the power of the frequency band is therefore estimated, and the desired modulation of this feature space is reinforced using appropriate visual, auditory or haptic feedback (Gharabaghi et al., 2014a,b). The feature weights are deliberately constrained during these interventions and the modality of feedback is designed to maximize the reinforcing effect of NFB (Sherlin et al., 2011; Vukelić et al., 2014). By contrast, during assistive BCI, the feature weights are calculated so as to allow maximal separation (Blankertz et al., 2008; Theodoridis and Koutroumbas, 2009) and the classification output is used for communication or robotic control (Wolpaw et al., 2002). While the primary goal for assistive BCI is accuracy and speed, the main goal for restorative BCI is reinforcement and learning. A theoretical difference therefore exists between the self-regulation of brain activity and the classification algorithm (Wood et al., 2014). However, although there are several ways of measuring the performance of an assistive BCI (Thomas et al., 2013; Thompson et al., 2013), similar appropriate measures for restorative BCI and NFB are currently not available. The most common measure for BCI is classification accuracy (CA). While the magnitude of CA has been used to estimate subject’s ability to perform brain self-regulation, this interpretation currently lacks theoretical foundation (Blankertz et al., 2010; Buch et al., 2012; Hammer et al., 2012). What is more, there is no consensus regarding what approaches are appropriate for disentangling the performance of subject and classifier from each other, nor is there any theory as to how they are connected with each other.

By integrating classification theory (Theodoridis and Koutroumbas, 2009) with item response theory (Safrit et al., 1989), we describe how the relationship between the classification algorithm and the ability for self-regulation can be understood. In addition, on the basis of the theory of cognitive load for instructional design (Sweller, 1994; Schnotz and Kürschner, 2007), we will describe how the CA can be interpreted within the framework of NFB training. Our argument is based on the fact that it is possible to make an off-line calculation of the positive rates for different classifiers and thresholds. We will argue that the true positive and the false positive curve provide information about the subject’s ability and his/her performance when support is provided. Moreover, since the shape of CA depends on the difference between true and false positive rate (FPR), we propose that it contains information about the subject’s zone of proximal development (ZPD). Therefore, on the basis of the theory of cognitive learning, the ZPD may serve as an indirect measure of the subject’s cognitive resources (Allal and Ducrey, 2000; Schnotz and Kürschner, 2007).

In this respect, the goal of this paper is to provide a measurement theory for subjects’ abilities and ZPD during NFB training. We support this theory by mathematical models and by evidence from empirical data.

## Empirical dataset

Exemplary data is based on two right-handed, healthy subjects, one female (age 19) and one male (age 31), who presented different abilities for brain self-regulation. They each performed 75 trials of cued motor imagery. The trial structure consisted of consecutive preparatory (2 s), motor imagery (6 s) and rest (6 s) phases, each of which was initiated by a specific auditory cue. Electroencephalography (EEG) was recorded at 64 channels in accordance with the 10–20 system with Brain Products amplifiers and analyzed offline with custom-written scripts and Fieldtrip in Matlab (Oostenveld et al., 2011) according to the following steps. Data was down-sampled to 200 Hz and band-pass filtered between 14 and 26 Hz using a Butterworth filter. Wavelet transformation was used to perform a time-frequency analysis for time steps of 50 ms for the power in the β-range (15–25 Hz) over sensorimotor regions (FC3, C3 and CP3). For each trial, the power at each time point was normalized by z-scoring based on the mean and standard deviation of the power distribution in the rest and preparatory phase. Both subjects gave written, informed consent prior to participation. The study was approved by the local ethics committee.

## Linking subject’s ability for brain self-regulation with the classification performance

In the following section, we will propose a link between the ability for brain self-regulation, as estimated by the item function, with the classifier performance, as estimated by the rate function. This integration will enable us to apply off-line analysis of the positive rate across different thresholds to determine the subjects’ ability for brain self-regulation.

### Rate functions

When applying NFB for therapeutic purposes, a two-class separation of brain states is usually performed, i.e., rest vs. learned self-regulation of brain activity. Due to the fact that most of the classifiers used in NFB are based on supervised learning algorithms employing linear discriminant analysis, the sensitivity and specificity of the classifier can be calculated relatively easily. The sensitivity informs us how often the classifier detects sufficient self-regulation while the subject is performing brain self-regulation (true positive rate or TPR). The specificity informs us how often the classifier detects rest while the subject is performing insufficient brain self-regulation (true negative rate or TNR). Since the probabilities of each conditional classification must add up to 1 within each class, the false negative rate (FNR) is equal to 1-TPR, and the FPR is equal to 1-TNR. CA is based on the average of TPR and TNR.

$$CA=\frac{\left(\text{TPR}+\text{TNR}\right)}{2}$$

### Threshold-based rate functions

**Figure 1:**
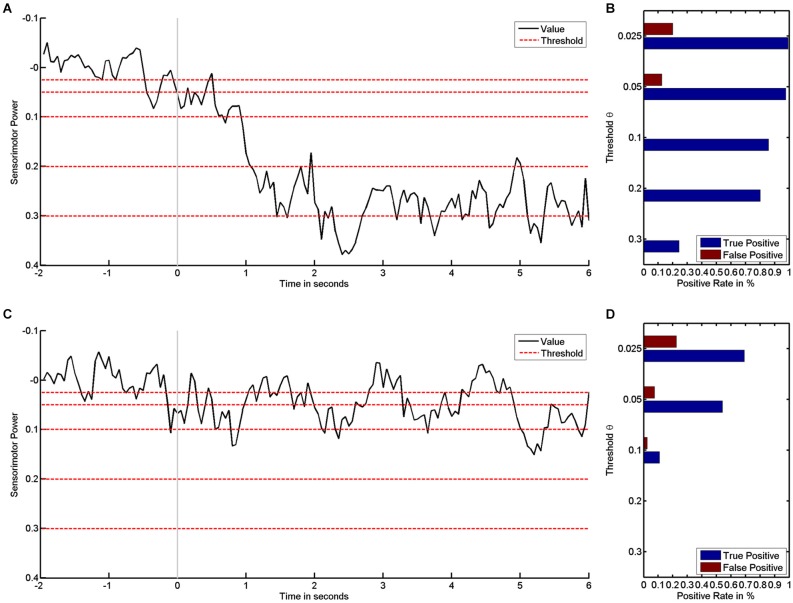
**It shows how sensorimotor beta power values are transformed into positive rates by threshold**. Left subplots show time course of sensorimotor power in black and different threshold levels in red for subject #1 **(A)** and subject #2 **(C)**. The respective rates of false and true positives for the first **(B)** and second subject **(D)** show how rates decrease as the threshold increases.

These rates are functions of the threshold θ (Theodoridis and Koutroumbas, 2009). During the training, the threshold θ usually remains fixed, and the rates therefore also remain fixed. However, provided that the electrophysiological signals have been recorded and stored, the positive rate can be calculated offline after the training for any threshold. We exemplify this by the empirical dataset (see Figure 1): the higher—i.e., the more challenging—the threshold, the stronger the desynchronization must be if it is to be classified as positive (see Figure 1A). The examples also reveal how subjects vary in their ability for brain self-regulation, e.g., sensorimotor beta-band desynchronization. The first subject shows stronger desynchronization and is thus able to reach more challenging thresholds (see Figure 1A) than the second subject, who has less pronounced brain self-regulation (see Figure 1C).

In parallel, this data enables us to characterize the classifier performance: detecting “positive” during the motor imagery phase (second 0 to 6) is a true positive, whereas “positive” during the preparatory phase (second −2 to 0) is a false positive. The average rate of positives for each phase is a suitable measure for characterizing a classifier’s performance, i.e., true and FPRs are expressed as probabilities in the range between 0 and 1 (see Figures 1B/D). The first subject has higher TPRs for all thresholds (see Figure 1B) than the second subject (see Figure 1D).

**Figure 2:**
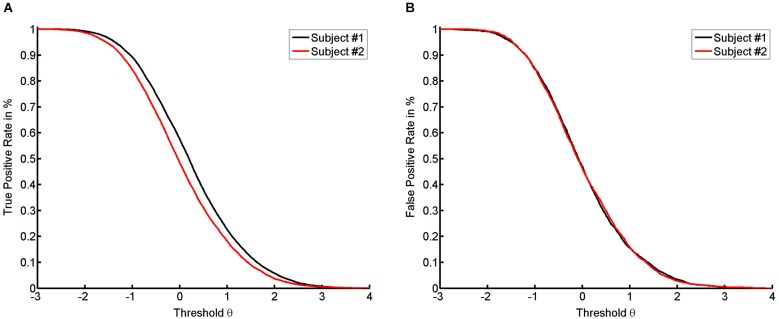
**It shows the numerical results with true positive rate (TPR) (A) and false positive rate (FPR) (B) for both subjects on the basis of the average across trials**.

For most classifiers, the rate functions result in sigmoidal rate curves. We show this sigmoidal shape for the TPR of the empirical dataset (see Figure 2A). The higher the thresholds, the more the probability of success decreases in a logistic fashion. Accordingly, the location of the first subject’s true positive curve is further to the right, indicating a generally higher success rate. However, the shape of the respective curves for the first and the second subject are highly similar.

### Item functions

The *rate functions* described resemble the *item functions* used in the psychometric item-response theory (IRT). In various fields of research, such as in assessment psychology (De Champlain, 2010) or motor behavior research (Safrit et al., 1989), the parameters of the item functions are usually estimated across datasets of several subjects and items. This enables us to quantify the respective variability of subject’s ability and task difficulty necessary for fitting algorithms. If, for example a mathematical test battery is distributed to a school class, the marginal success rates enable us to estimate the difficulty of a specific test and the ability of a single subject. Generally speaking, students with a higher mathematical ability achieve a higher success rate, and easier tests should result in higher average success rates. This information about the relative ranks based on success rates can be used for parameter estimation. More specifically, the shape of these success curves can be approximated by a two-parameter logistic model (2PLM) using the following function (Safrit et al., 1989; De Champlain, 2010):

$$P=\frac{1}{1+e^{-D(\alpha -\theta )}}$$

In this function, *e* is Euler’s number and D is the slope of the curve. The parameter θ, generally known as the threshold parameter in rate functions, now represents the difficulty of the task. Ability α and difficulty θ are located on the same axis. They can therefore be measured in one dimension. The location of the curve depends on the difference between the difficulty of a task and the subject’s ability α. The shape of the curve depends on the slope parameter D, and a value of ~1.7 would result in an approximate fit for a normal distribution (De Champlain, 2010). If the slope is identical for all curves, all item function are parallel and the difficulty level θ is the only changeable parameter.

### Similarity of rate and item functions

For dichotomous items, the probability of success is modeled as a function of the difficulty of the item and the ability of the subject. Assuming that the latter is stable, the difficulty of an item then depends on the design of the task. The combination of NFB task, i.e., the classification algorithm, the trial structure, the cues and any instructions or extraneous aspects constitute the phrasing of such an item. By way of example: in NFB training, our aim is to differentiate between sufficient and insufficient modulation of brain activity. If we were to use a questionnaire instead of EEG and BCI, we might phrase an item: “Are you currently performing sufficient brain self-regulation?” to which the possible answers would be “yes” and “no”. However, since the decision about “yes” or “no” is based on physiological recordings during the task, a *post hoc* reassessment for different thresholds is possible. This recalculation enables us to apply virtually the same items over a range of difficulties. In addition, threshold-based recalculation modifies only one aspect of the “item”, namely the difficulty parameter; an aspect that lies in one clearly defined dimension. These properties (uni-dimensionality, off-line analysis) allow for an interpretation of the parameters that describe the positive rate curves on the threshold dimension within the framework of the item response theory.

## Interpretation of curve parameters

### True positive curve reports on ability

Due to the fact that the measurement of the subject’s ability for brain self-regulation can be performed *post hoc*, NFB training is comparable to an action like videotaping a sports exercise such as long jump. The data acquired during the task can then be used later to estimate several aspects of the performance. As in sports training, the compound ability can be divided into sub-sets (e.g., sprinting, take-off, and landing in long jump). In this example, the coach would be ill-advised to reward any jump independent of the actual performance. Therefore, specificity matters.

NFB training has the ability to provide this specificity by selecting the appropriate features and classification algorithms. In addition to determining the threshold to be passed by the event-related desynchronisation (ERD), additional features might include the speed of the power dip in the first two seconds or continuity of desynchronization. This highlights the fact that the reinforced features must be carefully selected for their respective clinical or rehabilitative purpose.

In this respect, it is also important to note that functional improvement is a combination of several abilities and preconditions, of which for instance, brain self-regulation of sensorimotor beta-rhythms is only one example. Others, such as reaching out and holding a certain position with the upper limb, as assessed in the Fugl-Meyer assessment (Deakin et al., 2003), or interacting with an object, as assessed in the Broetz hand assessment (Brötz et al., 2014), necessitate the involvement of parieto-frontal circuits for motor planning (Andersen and Cui, 2009) and sensorimotor circuits for execution (Chouinard and Paus, 2006). Along these lines, stroke survivors who train to modulate the activity of the primary motor cortex (Kaiser et al., 2011; Kilavik et al., 2012) show improvements in this ability only if the fronto-parietal integrity is preserved (Buch et al., 2012). Training such ability of brain-self-regulation may therefore be related both directly and indirectly to the respective function, e.g., moving the upper extremity. However, improving brain self-regulation does not necessarily lead to functional improvements, since these may also depend on abilities and preconditions that are not influenced by the NFB training. Improvements are therefore required with regard to the clinical efficacy of such a training (Ramos-Murguialday et al., 2013; Ang et al., 2014) by researching the feedback modality (Gomez-Rodriguez et al., 2011) or using it in combination with simultaneous cortical stimulation (Gharabaghi et al., 2014a). Screening examinations might also be necessary to determine the eligibility of subjects for a specific intervention (Stinear et al., 2012; Burke Quinlan et al., 2014; Vukelić et al., 2014). In addition, the validity of functional assessment scores requires re-evaluation in the light of biomarkers of sub-clinical improvement.

We therefore conclude that the location of the TPR can be interpreted as a subject’s ability for brain self-regulation, regardless of the potential influence of this ability on a specific function. In this respect, the location of the true positive curve—mathematically speaking, the point of maximal slope and halfway between success and failure—provides information about the subject’s ability to perform the task which is defined by the features and the classifier.

### False positive curve reports on attempt

**Figure 3:**
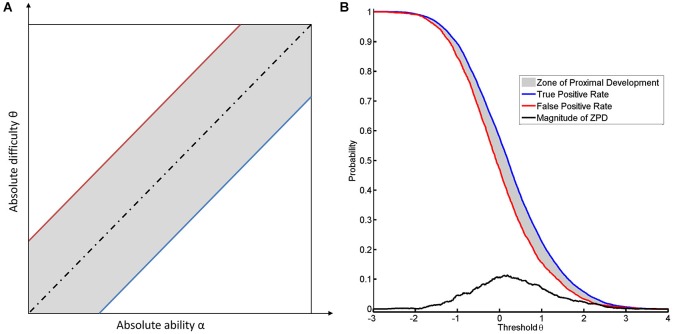
**It illustrates the concept of ZPD. (A)** shows the dependance of the location of the ZPD from the absolute difficulty and the absolute ability. The ZPD width is based on the between the true and false positive rate. The blue line indicates the success rate of the task when performed without help (true positive rate) and the red line indicates the success rate due to help (false positive rate). The dotted black line indicates the equality of difficulty and ability. The area of ZPD is shown in gray. **(B)** shows the ZPD based on FPR and TPR over different thresholds for the first subject.

According to our previous example, a long jump coach would be ill-advised not to reward any jump. To be more precise, for reasons of motivation, even attempts should sometimes be rewarded, or support is required to transform an attempt into a success. In the case of NFB, specificity and sensitivity also have to be balanced according to their importance for learning. If the task remains identical, such a balance can only be achieved by changing the threshold. Decreasing the threshold increases the number of false positives (see Figure 2B). Since the classifier normalizes to rest, there is no apparent difference in the location of the FPR between the two subjects (see Figure 2B). This indicates that the subjects have the same opportunity to try to perform the task. This theory is supported by the following line of argument. In this context, “support” or “help” can be formalized by assuming that the subject with the current level of ability α is unable to perform the task at the given level of difficulty θ, whereas providing help will lead to success. If we then detect a success, this will be a “false positive” result, since the subject’s current ability is too low for him/her to actually succeed. If no help is provided, the success achieved will be a “true positive” result. This approach will lead to a range of thresholds which are defined by two limits. The lower limit will be marked by the most difficult task that the subject can perform when help is provided. The upper limit is defined by the most difficult task that can be performed by the subject without help (see Figure 3A). Once the subject no longer benefits from help, e.g., due to overly high intrinsic or extrinsic load, he can no longer benefit from the training.

### Shape of classification accuracy sheds light on the zone of proximal development

The range between the most difficult tasks that can be achieved by the subject with and without help, respectively, can be defined as the ZPD. Cognitive load theory argues that mental load can be divided into three categories: intrinsic load, extrinsic load and germane load (Jong, 2009). Intrinsic load resembles the difficulty of the task and is mainly caused by the element interactivity of the task. Extraneous load is mainly caused by irrelevant information. Germane load is caused by the construction and subsequent automation of schemas, i.e., learning. Lower difficulty results in a reduction of intrinsic load while extrinsic load will increase, since the instructional material now contains irrelevant information. If a task is too easy (i.e., θ ≪ α) or too difficult (i.e., θ ≫ α) for a given ability, the extrinsic or the intrinsic load of the task would be too high.

For every given level of difficulty and ability for a task, there is therefore a ZPD, where learning is possible (Schnotz and Kürschner, 2007). The cognitive load theory thus provides a feasible explanation as to why the boundaries of ZPD are characterized by TPR and FPR (Allal and Ducrey, 2000; see Figure 3B). A second line of argumentation considers the likelihood of reward. Since a subject cannot discern a reward with identical qualities, the only way of differentiating between a true and a false reward is to determine the relative probability of their occurrence. The difference between the true and FPR might therefore be a good approximation of the difference with respect to the informational content of the two reward rates. Although more elaborate measures might be better suited to this divergence (MacKay, 2003), the most straightforward approach would consider the magnitude and the shape of ZPD as estimated by a linear transformation of CA in accordance with the following equations:

$$CA=\frac{TPR+TNR}{2}$$

ZPD=TPR−FPR

TNR=1−FPR

$$CA=\frac{TPR+(1-FPR)}{2}=\frac{TPR-FPR+1}{2}$$

(2*CA)−1=TPR−FPR

ZPD=(2*CA)−1

## Conclusion and outlook

In the sections above, we have shown how the true and false positives rates of brain self-regulation can be interpreted within the framework of NFB. We have demonstrated that there is a natural relationship between classification of rate functions and item response functions. We have revealed the connection between applying a threshold to ERD signals and estimating the ability to perform ERD. In this respect, the true positive curve provides information as to the brain’s ability to perform brain self-regulation in a NFB task. In addition, we showed that not only can the false positive curve provide information about attempts to perform the task but it can also set the lower limit of the ZPD. Below, we will illustrate how the ZPD, in its capacity as a transformation of CA, can support the instructional design of NFB interventions.

### Conclusion regarding classification accuracy

**Figure 4:**
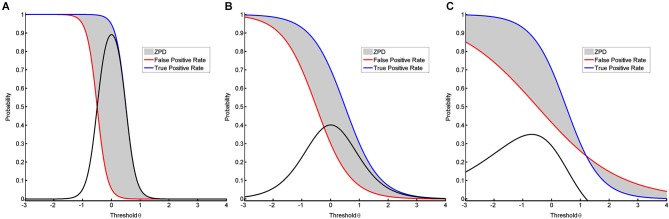
**It visualizes different shapes of ZPD.** In the first two models **(A, B)**, the discrimination is different despite the distance between the two conditions being equal, resulting in ZPDs with equal areas. **(A)** shows success rates for a peaky but narrow ZPD based on a two-parametric model with equal but high discrimination values for both positive functions. **(B)** shows success rates for a broad but flat ZPD based on a two-parametric model with equal but low discrimination values. **(C)** shows success rates for a ZPD with a break-point based on a two-parametric model with unequal discrimination values.

The ZPD can be used to compare different classification algorithms. In BCI approaches, classification algorithms are often trained to maximize CA. This can result in a peaky but narrow ZPD (see Figure 4A) instead of a flat but broad ZPD (see Figure 4B), although the area of ZPD is equal in both cases. A more broadly shaped ZPD indicates that learning can occur over a larger range of thresholds, whereas a peaky shape means that maximal help is available only for a narrow range of thresholds. This being the case, slight misalignments might have significant adverse effects. The shape of CA may therefore serve as a measure to evaluate whether or not a NFB task is instructionally effective. While the best general instructional efficacy is obviously achieved by NFB training with a high *and* broad ZPD, interpreting the shape enables us to apply tailored approaches. These might be more effective with regard to instructional needs for specific subjects and environments. A broad ZPD might be more robust for home-based training with low availability of supervision and the possibility of noisy measurements. A peaky ZPD might be more suitable for environments where professionals can perform alignments, i.e., adapt the classification algorithm or correct noisy measurements. Shaping the ZPD can thus support instructional design of NFB interventions. The approach presented here will also be applicable to classification algorithms resulting in non-normal distributions where TPR and TNR are calculated numerically (see Figure 3B), since the interpretation is also supported by non-parametric IRT-models provided that TPR and FPR are monotonic functions (Mokken and Lewis, 1982; Rost, 2004).

### Relationship to alternative measures of performance and feedback

CA is by far the most widely reported measure of performance for BCIs and can be used for both synchronized (e.g., cued) and self-paced interventions (Thomas et al., 2013). However, for some clinical applications, additional measures of performance that are regarded as relevant for the treatment goal have been developed. These include the latency to movement onset or the maximum consecutive movement time in stroke rehabilitation (Ramos-Murguialday et al., 2013) and the path efficiency for a high degree of freedom prosthetic control in tetraplegia (Collinger et al., 2013). How does the ZPD now relate to these alternative performance measures?

These alternative measures may sometimes be translated into one of the basic measures used for calculating the ZPD, e.g., the average movement rate could also be understood as a TPR. However, such measures are much more liable to contain unique information about additional abilities that are required for the given task to be performed, such as already mentioned in the paragraph on the interpretation of the true positive curve.

Since learning is conceptually linked to the accuracy of feedback, we propose that a NFB task should be characterized by its instructional efficacy with regard to the action to be trained. This instructional efficacy is characterized by the feedback curves. In this context, the CA changes as soon as the coupling of the feedback to the action changes. The shape of the ZPD will therefore be useful for the instructional design of the intervention and tends to be independent of other task-specific measures. If, for example, the subject receives feedback to alternative actions, any improvement in these actions will be caused by the task’s instructional design. In this respect, a ZPD, e.g., for latency of movement onset or path efficiency, may also exist. Estimating the ZPD for these measures would be similar to the approach illustrated above.

It should be borne in mind that the theory presented here is based on the classical binary feedback, the distance between feedback and no feedback being one bit of information, i.e., reward (Ortega and Braun, 2010). Alternative approaches such as continuous feedback (e.g., the frequency of an auditory signal) or psychophysical perception rules (e.g., the perception of the duration of binary feedback in a log-linear fashion) do, of course, affect the bit-rate and may thus increase the achievable speed of learning. However, since the ZPD is based on a single bit as a distance metric, adequate mapping of the ZPD for alternative feedback approaches will probably be mathematically demanding. In order to interpret the curves under such conditions, further research and specific transformations might be necessary. A system analytical perspective, where continuous feedback can be understood as a pattern of step functions, and a complex-valued ZPD might help to solve such aspects. Nonetheless, the real-valued ZPD based on the single bit of feedback will also remain a fundamental building block of such advanced approaches.

### Conclusion with regard to cognitive resources

The ZPD may also act as a measure to compare different subjects with regard to their cognitive resources for a NFB task. If two subjects perform the very same NFB training, one might show a peaky and narrow ZPD (see Figure 4A) while the other has a broad and flat ZPD (see Figure 4B). Since in this case both curves indicate equal abilities, this difference in the respective shapes requires an alternative explanation. On the basis of the relevance of cognitive resources for the ZPD (Schnotz and Kürschner, 2007), we postulate that the shape of ZPD can also be applied to measure a subject’s cognitive resources for coping with the mental load that occurs during a misalignment between ability and difficulty. Such an interpretation would, furthermore, permit a different view on the discussion about BCI illiteracy (Vidaurre and Blankertz, 2010). In particular, when the curves of TPR and FPR cross, they provide information about the specific break-points of that task. At this point, any support provided by the instructional design of the training will cease to be beneficial and will begin to be detrimental for the performance (see Figure 4C). This would be indicated by a negative value for the ZPD.

However, these concepts require validation by future research. Measurements of cognitive resources are currently based on psychophysiological recordings (e.g., heart rate variability, blink rate, electrodermal response), which are highly variable and very difficult to generalize across task conditions (Cegarra and Chevalier, 2008; Novak et al., 2010). Motor imagery itself can also cause vegetative effects related to the imagined movement, e.g., subjects imagining running at 12 km/h had an increased heart rate and pulmonary ventilation as compared to walking at 5 km/h (Decety et al., 1991). Mental imagery might therefore affect psychophysiological biomarkers, masking the measurement of the mental effort unrelated to the imagery content. One alternative to psychophysiological measures is the application of self-rating questionnaires. However, from the subject’s point of view, it is often not possible to distinguish between the intrinsic, extrinsic and germane load (Cegarra and Chevalier, 2008). What is more, many psychophysiological measures and questionnaires can be sampled only at a very low rate. For example, the low frequency part of the heart rate commences at 0.04 Hz (Malik et al., 1996), meaning that at least 25 s of clean data have to be recorded for adequate frequency resolution of the Fourier transformation. Furthermore, slow frequency fluctuations in the EEG (<0.1 Hz) can correlate with psychophysiological performance, but they require similarly large time windows. In addition, slow fluctuations in the EEG measurements appear to be highly masked by imagery-related fluctuations, e.g., movement-related cortical potentials (Shibasaki and Hallett, 2006). This is also an issue if higher frequency components of the EEG are used to estimate cognitive resources, e.g., in the gamma range (Grosse-Wentrup et al., 2011), since they need to be disentangled from pure motor-related fluctuations in the same frequency band (de Lange et al., 2008; Miller et al., 2012).

In this context, the shape of ZPD might prove useful for disentangling the multitude of interacting and complex psychophysiological measurements in challenging tasks. This perspective is in agreement with the understanding that a proper alignment of ability and difficulty will reduce mental effort (Schnotz and Kürschner, 2007). Future studies might focus on psychophysiological correlates of the shape of the ZPD. Furthermore, improving the instructional material should help to reduce extrinsic load. NFB training could similarly be supported by “instructions”, e.g., by providing haptic feedback (Gomez-Rodriguez et al., 2011) or visual and auditory cueing (Heremans et al., 2009). Systematic research on the impact of these feedback modalities on the ZPD might provide insight on their utility in guiding instructional design.

## Conflict of interest statement

The authors declare that the research was conducted in the absence of any commercial or financial relationships that could be construed as a potential conflict of interest.
